# A Strategy for Screening the Lipid-Lowering Components in *Alismatis Rhizoma* Decoction Based on Spectrum-Effect Analysis

**DOI:** 10.1155/2022/2363242

**Published:** 2022-01-04

**Authors:** Xiao-Yan Chang, Jia-Shuo Wu, Fang-Qing Zhang, Zhuang-Zhuang Li, Wei-Yi Jin, Jing-Xun Wang, Wei-Hua Wang, Yue Shi

**Affiliations:** ^1^Institute of Medicinal Plant Development, Chinese Academy of Medical Sciences & Peking Union Medical College, Beijing 100193, China; ^2^Hebei Medical University, Shijiazhuang 050017, China; ^3^Tsinghua University, Beijing 100084, China

## Abstract

*Alismatis Rhizoma* decoction (ARD), comprised of *Alisma plantago-aquatica subsp. orientale* (Sam.) Sam and *Atractylodes macrocephala* Koidz. at a ratio of 5 : 2, is a classic traditional Chinese medicine (TCM) formula with successful clinical hypolipidemic effect. This paper aimed to explore the major bioactive compounds and potential mechanism of ARD in the treatment of hyperlipidemia on the basis of spectrum-eﬀect analysis and molecular docking. Nine ARD samples with varying ratios of the constituent herbs were prepared and analyzed by UPLC-Q-TOF/MS to obtain the chemical spectra. Then, the lipid-lowering ability of the nine samples was tested in an oleic acid-induced lipid accumulation model in human hepatoma cells (HepG_2_). Grey relational analysis and partial least squares regression analysis were then performed to determine the correlation between the chemical spectrums and lipid-lowering efficacies of ARD. The potential mechanisms of the effective compounds were investigated by docking with the farnesoid X receptor (FXR) protein. The results indicated that alisol B 23-acetate, alisol C 23-acetate, and alisol B appeared to be the core eﬀective components on hyperlipidemia in ARD. Molecular docking further demonstrated that all three compounds could bind to FXR and were potential FXR agonists for the treatment of hyperlipidemia. This study elucidated the eﬀective components and potential molecular mechanism of action of ARD for treating hyperlipidemia from a perspective of different compatibility, providing a new and feasible reference for the research of TCM formulas such as ARD.

## 1. Introduction

*Alismatis Rhizoma* decoction (ARD), comprised of the rhizomes of *Alisma orientale* (Sam.) Juzep. (AR) and the rhizomes of *Atractylodes macrocephala* Koidz. (AMR) at a ratio of 5 : 2, was first described by Zhongjing Zhang in “Jingui Yaolue” [1]. Although the composition of ARD is simple, its therapeutic effect is remarkable. Recent studies show that ARD can reduce the triglyceride and total cholesterol levels in serum and liver and significantly increase the concentrations of serum HDL-cholesterol, possessing successful clinical capability of treating hyperlipidemia [2, 3]. Even though the hypolipidemic activity of ARD is notable, little information is available about the active compounds in ARD, obstructing the in-depth study of the molecular mechanism of ARD for the treatment of hyperlipidemia. So far, triterpenoids, sesquiterpenoids, ﬂavonoids, and polysaccharides have been identified in the two individual herbs AR and AMR that exhibit hypolipidemic, antioxidative, and anti-inflammatory effects [4–7]. However, the core hypolipidemic components of ARD remain to be clarified.

As a highly complex system containing multiple components, rational strategies are required to be devised to identify pharmacologically active compounds in TCM. For instance, the spectrum-effect study is increasingly being used to discern the speciﬁc biological active compounds in TCM by analyzing the correlation between the spectral profiles and biological effects [8]. Reasonable chemometric methods, such as canonical correlation analysis, grey relational analysis, and partial least squares regression analysis, are frequently utilized for the spectrum-effect research [9]. Also, multiple data analysis methods are recommended to be simultaneously applied into the process of exploring the active ingredients to obtain better accuracy. Furthermore, for the reported literature related to spectrum-effect study, most of them focused on single herb medicine, and herbal formula as the research object only accounted for a small part due to its complicated composition. So, developing appropriate and feasible approaches from a holistic perspective to carry out the spectrum-effect study of TCM prescription possesses important practical significance.

The farnesoid X receptor (FXR), highly expressed in the liver, kidney, intestines, and adrenal glands, is a bile acid receptor, playing considerable roles in the homeostasis of cholesterol and bile acid [10, 11]. Therefore, regulators of FXR are promising therapeutic agents against hyperlipidemia, cholestasis, atherosclerosis, and liver fibrosis [12–14]. There is considerable interest in developing novel selective FXR ligands to treat diseases related to disorders of lipid metabolism. It has been reported that ARD could activate the LXR*α*/ABCA1 pathway and maintain homeostatic balance of ABCA1, which played a role in promoting lipid metabolism [15]. Moreover, the chemical components in ARD such as alisol M 23-acetate, alisol A, and alisol B 23-acetate were reported to act as the LXR agonist [16, 17]. Furthermore, the HepG_2_ cell line can express FXR, and the cells are often used to investigate the agonists or inhibitors of FXR [18, 19]. The HepG_2_ cell line also has the advantages of easy culture and transfection, and most features of the hepatocyte cell line are preserved in HepG_2_ cells.

In the present study, we started from changing the compatibility of AR and AMR in ARD to comprehensively implement the spectrum-effect study. The chemical spectra and lipid-lowering efficacies of ARD extracts in HepG_2_ cells with different compatibilities were correlated to reveal the core lipid-lowering compounds, and their potential interactions with FXR were then determined by molecular docking in order to verify the accuracy of spectrum-effect analysis and probe the mechanism of action of ARD on hyperlipidemia.

## 2. Materials and Methods

### 2.1. Reagents

AR (Batch number: DD6081) and AMR (Batch number: DD8061) were purchased from Beijing Huamiao Pharmaceutical Co. Ltd. (Beijing, China) and their quality was in accordance with the Chinese Pharmacopoeia (2015 Version). The alisol B, alisol B-23 acetate, alisol C, alisol C-23 acetate, and atractylenolide I were purchased from Chroma-Biotechnology Co. Ltd. (Chengdu, China). Honeywell Burdick & Jackson Company (NJ, USA) provided HPLC-grade methanol and acetonitrile, and formic acid (MS grade) was from Fisher Scientific (Spain). Deionized water was obtained using the Milli-Q system (Millipore, MA, USA). Cell culture reagents, and the Nile red dye and cell counting kit-8 (CCK-8 kit) were respectively purchased from Thermo Fisher Scientific (MA, USA) and Beijing Solarbio Science & Technology Co. Ltd. DMSO and oleic acid were from Sigma-Aldrich (MO, USA). Other compounds were of analytical grade.

### 2.2. Preparation of Samples

Nine different ARD samples were prepared by combining AR and AMR in the ratio (w/w) of 3 : 1, 5 : 2, 2 : 1, 3 : 2, 1 : 1, 2 : 3, 1 : 2, 2 : 5, and 1 : 3. The samples were crushed and refluxed thrice in 95% ethanol for 2 h each. The extracts were filtered, combined, and concentrated to obtain a 10% ethanol extract. To prepare working solutions for chromatography, 50 mg of each ARD crude extract was diluted in 10 mL 80% methanol to yield a solution of concentration 5 mg/mL. The standard solution was prepared by dissolving accurately weighted portions of the five reference compounds in MeOH.

### 2.3. UPLC-MS Analysis

#### 2.3.1. UPLC-MS Conditions

The ARD and standard solutions were centrifuged at 10,033 g for 10 min, and 2 *μ*L aliquots were respectively injected into the Waters ACQUITY UPLC HSS T3 column (1.7 *μ*m × 2.1 mm × 100 mm; Waters, MA, USA) at 15°C in a Waters ACQUITY UPLC system. The samples were eluted with the flow rate at 0.2 mL/min using acetonitrile (solvent A) and 0.1% v/v formic acid in water (solvent B). The linear gradient was as follows: 0–10 min, 0%–1% A; 10–15 min, 1%–3% A; 15–20 min, 3%–10% A; 20–25 min, 10%–15% A; 25–30 min, 15%–20% A; 30–35 min, 20%–30% A; 35–40 min, 30%–60% A; 40–45 min, 60%–80% A; 45–50 min, 80%–90% A; 50–55 min, 90%–100% A; and 55–60 min, 100%–0% A.

The mass spectra of the above eluents were acquired on the Waters SYNAPT G2 HDMS system (Waters Corp., USA) in positive ion mode by scanning over the m/z range of 50–1200. The following conditions were used: desolvation gas flow at 800 L/h and 450°C, cone gas flow at 50 L/h and 100°C, capillary voltage 3 kV, cone voltage 40 V, and scan time 0.5 s. Leucine enkephalin with an [M+H]^+^ ion at m/z 556.2771 was used as the lock mass. The Mass Lynx V 4.1 software was used for data analysis.

#### 2.3.2. The Establishment of the Spectrum

Under the abovementioned UPLC-MS conditions, the bask peak ion (BPI) chromatograms of the nine ARD samples were obtained as the chemical spectrum in the spectrum-effect study. Then, nine chemical chromatograms were automatically matched by the professional software named Similarity Evaluation System for Chromatographic Spectrum of Traditional Chinese Medicine (2004A version, Committee of Chinese Pharmacopeia) to generate the common peaks of the different ARD samples [20].

#### 2.3.3. Qualitative Analysis of ARD

The UPLC-MS-based qualitative analysis of ARD was performed in our previous study. The chromatographic and mass spectrometric conditions were the same as the conditions above. The results could be confirmed in the published paper in detail [21].

### 2.4. Lipid-Lowering Effect Investigations

#### 2.4.1. Cell Viability Assay [21]

HepG_2_ cells from the laboratory of Chinese Academy of Medical Sciences and Peking Union Medical College (Beijing, China) were maintained in complete DMEM (10% FBS, 100 units/mL penicillin, and 100 *μ*g/mL streptomycin) at 37°C under 5% CO_2_. The cells were seeded in a 96-well plate at the density of 1 × 10^4^ cells/well and cultured for 24 h with 10, 20, 40, 80, 160, 320, and 640 *μ*g/mL ARD. After washing thrice with PBS, fresh medium with 100 *μ*L 10% CCK-8 solution was dispensed per well, and the cells were incubated for another hour [22]. The absorbance at 450 nm was measured. The experiment was repeated at least thrice, with six replicates per dose.

#### 2.4.2. Inhibition of Lipid Accumulation in HepG_2_ Cells of ARD Samples

The HepG_2_ cells were seeded in a 96-well cell plate at the density of 1 × 10^4^ cells per well and cultured for 24 h. After replacing with fresh medium supplemented with 0.2 mM oleic acid and the suitable ARD dosage, the cells were incubated for 24 h [20, 21, 23]. After washing thrice with PBS, the cells were stained with 0.3 mM Nile red for 15 min in the dark and rinsed with PBS to remove the excess dye [24, 25]. The fluorescence emission at 572 nm was read using a microplate reader (Tecan, Switzerland).

#### 2.4.3. Statistical Analysis

All the data were analyzed by one-way analysis of variance (ANOVA) using SPSS 23.0 (SPSS, USA). Results were expressed as mean ± standard deviation (S.D.). A value of *P* < 0.05 was considered to be a signiﬁcant difference.

### 2.5. Spectrum-Effect Analysis

#### 2.5.1. Grey Relational Analysis (GRA)

GRA, originating from grey system theory, is used to calculate the correlation between multiple variables on the basis of the geometric similarities [26]. Owing to its superiority in analyzing the complicated relationships of multiple variables, GRA is particularly suitable to analyze the mixture consisting of known and unknown information such as TCM which contains a myriad of chemicals [9]. For these reasons, the online software SPSSAU was utilized to synthetically evaluate the correlation between the common peaks from the chromatographic spectrums and lipid-lowering action to seek out the active compounds of ARD. In this analysis, grey relational coefficients were used to assess the degree of correlation. A high grey relational coefficient implies a high correlation between the peaks and the efficiency. Thus, peaks with high grey relational coefficients were selected [27, 28].

#### 2.5.2. Partial Least Squares Regression (PLSR) Analysis

PLSR is another data analysis technique which is frequently applied to determine the correlation between a set of dependent variables and a larger set of independent variables [29]. It can reflect the dependency of each common peak to the efficiency in terms of the variable importance in projection (VIP) value, and values greater than 1 indicate significant correlation [30]. Similarly, the regression coefficient is also an important parameter of PLSR used in the spectrum-effect relationship study to reflect the qualitative trend [31]. The PLSR between chemical spectrum and bioactivity was modeled using SIMCA-P 13.0 (Umetrics), and the regression coefficient and VIP values were calculated.

### 2.6. Molecular Docking

The MOE software was used for the docking between the ligands and the protein FXR. Before docking, the 2D structure of the small molecule compounds were converted to 3D structures through energy minimization. Then, the 3D X-ray structure of the protein FXR was downloaded from RCSB Protein Data Bank (PDB ID: 6HL1). In the MOE-Dock program, the force field of AMBER10: EHT and the implicit solvation model of Reaction Field (R-field) were selected for the docking. The side chains of the protein pocket were allowed to move according to ligand conformations during the docking process, and the weight used for tethering side chain atoms was set as 10. The docked positions were ranked by London dG scoring, and the top 10 positions were rescored by GBVI/WSA dG. The best binding conformation was one with least free energy.

## 3. Result

### 3.1. Establishment of the UPLC-MS Spectrum

#### 3.1.1. UPLC-MS Spectrums

**Figure 1:**
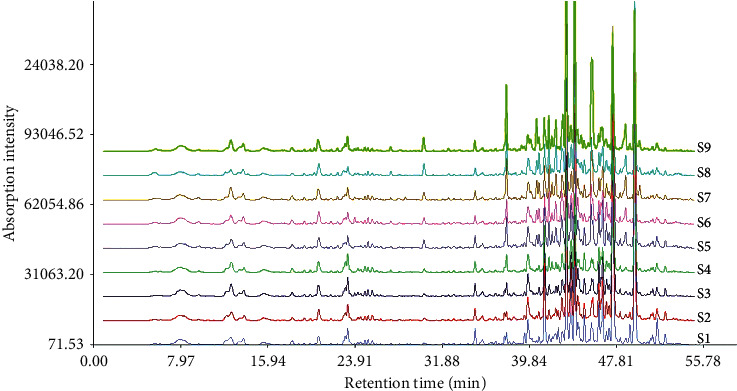
UPLC-MS spectra of the 9 ARD samples.

For the first step of spectrum-effect analysis, nine ARD samples with AR and AMR in the ratio (w/w) of 3 : 1, 5 : 2, 2 : 1, 3 : 2, 1 : 1, 2 : 3, 1 : 2, 2 : 5, and 1 : 3 were prepared and analyzed under the optimized UPLC-Q-TOF/MS analytical conditions. Nine MS spectra which represented the chemical information of ARD with different compatibility ratios were constructed (Figure 1). Then, the spectra were imported into the idiomatic software to automatically match the common peaks. Finally, based on retention time and the MS/MS spectra, a total of twenty common peaks were obtained as shown in Table 1. It was found that the nine samples possessed similar chemical profiles, but there were still some significant differences among these ARD samples due to the variations in the AR/AMR ratio, as values of the common peak areas fluctuated greatly in nine samples, reflecting unequal contents of the corresponding compounds. This result provided the essential content “spectrum” of the spectrum-effect study, and the calculated common peaks could be utilized to precisely pinpoint active ingredients in ARD.

#### 3.1.2. Identification of the Common Peaks

Among the twenty common peaks defined, peak X5, X9, X11, X18, and X20 were unambiguously identified as alisol C, alisol C 23-acetate, atractylenolide I, alisol B, and alisol B 23-acetates, respectively, by comparing their MS/MS spectra with the reference standards. Likewise, the peaks X3, X4, X6, X8, X10, X12, X14, X15, X16, and X17 were putatively determined as tetradecylcitric acid, 16-oxo-alisol A, 16-oxo-11-anhydro-alisol A, atractylenolactam, alisol L, alisol G, alisol M 23-acetate, alisol O, alisol I, and alisol L 23-acetate, respectively, based on previously reported literature information [32–36]. The retention time, name, formula, and MS/MS spectra of these compounds are summarized in Table 2. As for the other common peaks, their chemical structures needed to be confirmed by the help of additional analytical methods.

### 3.2. ARD Mitigated Lipid Accumulation in HepG_2_ Cells

As the chemical constituent basis is closely related to the pharmacological activity, examinations of the toxicities and activities of the nine ARD samples were performed as the second stage of the spectrum-effect study. As shown in Table 3, all nine ARD extracts at the concentration of 160 *μ*g/mL did not show significant impact on the cell viability of HepG_2_ cells, and this concentration was selected for the subsequent pharmacological assay.

An oleic acid-induced lipid accumulation model in HepG_2_ cells was utilized to evaluate the lipid-lowering ability of the nine ARD extracts, and Nile red staining was performed to quantify the intracellular lipid contents by detecting the fluorescence intensity. The lower the value of the fluorescence, the better was the eﬀect of the sample. When the nine ARD samples were added to the lipid accumulated model system, diﬀerent eﬃcacy values were obtained because of the different chemical substance basis (Table 3).

### 3.3. Combination of Suitable Mathematical Approaches for Relevance Analysis

#### 3.3.1. GRA

**Figure 2:**
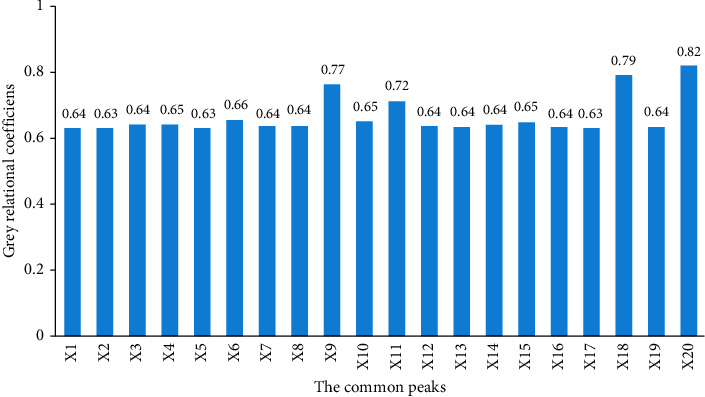
Grey relational analysis of the ﬁngerprint and bioactive effect. The grey relational coefficients were used to assess the correlation between the common peaks and the lipid-lowing efficiency. The common peaks and the grey relational coefficients were respectively used as X matrix and Y matrix to find the potentially active compounds.

The common peaks and the inhibition on lipid accumulation of ARD were, respectively, used as X matrix and Y matrix in the GRA to find the active compounds corresponding to the lipid-lowering efficacy. The result of the GRA was shown in Figure 2. The prominent peaks in terms of correlation coefficients were X20 (0.82), X18 (0.79), X9 (0.77), and X11 (0.72), which were respectively attributed to alisol B 23-acetate, alisol B, alisol C 23-acetate, and atractylenolide I, showing that these four peaks played major role in the activity in ARD. Furthermore, it could be seen that the whole four peaks had large peak areas, which were indicative of high contents in ARD. Thus, peak X20, X18, X9, and X11 were clearly pointed to major biological roles for the lipid-lowering effect.

#### 3.3.2. PLSR

**Figure 3:**
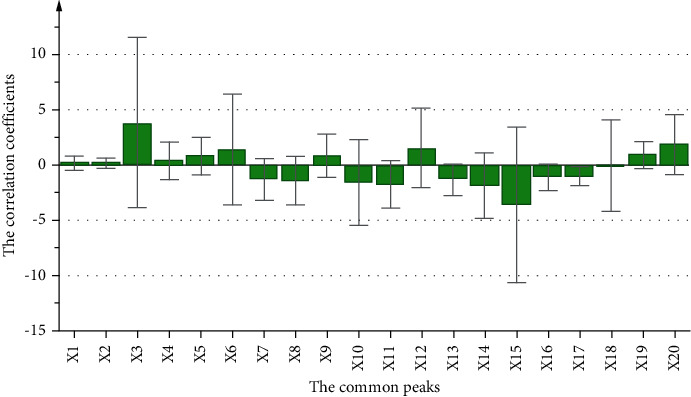
The correlation coefficients of the PLSR model.

In addition to GRA, the relationship between the twenty common peaks (X-variables) and the impact on lipid accumulation (Y-variables) was also evaluated by using a PLSR model. The R2X and Q2 values of the PLSR model were 0.997 and 0.986, respectively, demonstrating that the model was credible for the spectrum-effect analysis. As shown in Figure 3, the peaks X3 (aetradecylcitric acid), X6 (16-oxo-11-anhydro-alisol A), X12 (alisol G), and X20 (alisol B 23-acetate) correlated strongly with the lipid-lowering effect with high positive correlation coefficients.

**Figure 4:**
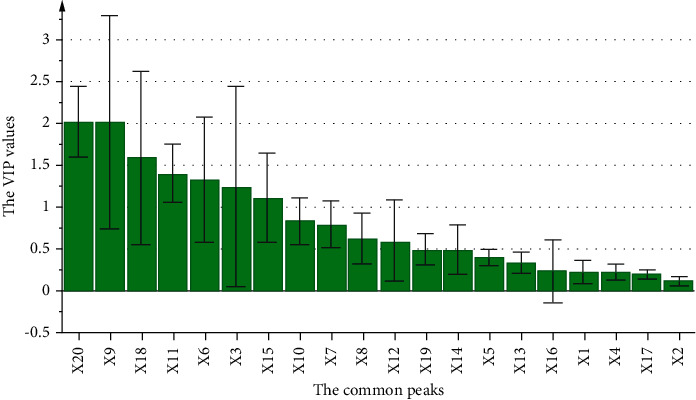
The VIP values of the PLSR model.

Furthermore, the VIP values (Figure 4) of peaks X20 (alisol B 23-acetate), X9 (alisol C 23-acetate), and X18 (alisol B) were over 1.5, showing these peaks possessed the most important eﬀects on the Y-variable. Also, the peaks X11 (atractylenolide I), X6 (16-oxo-11-anhydro-alisol A), X3 (aetradecylcitric acid), and X15 (alisol O) showed VIP values greater than 1, which were also indicative of biological significance. Based on the entire analysis of PLSR, peaks X20, X9, X18, X3, and X6 scored high in terms of VIP values and correlation coefficients and were eventually identified as the lipid-lowering active compounds in ARD.

Taken together the results from both the GRA and PLSR models, alisol B 23-acetate, alisol B, and alisol C 23-acetate were confidently identified as the major core lipid-lowering compounds in ARD.

### 3.4. Docking between the Effective Compounds and FXR

The MOE software was used for the docking between compounds alisol B 23-acetate, alisol B, alisol C 23-acetate, and protein FXR, and the binding sites and the docking scores were obtained. The docking scores of the above compounds are summarized in Supplementary Table S1.

Alisol B 23-acetate had the most negative docking score, which corresponded to best possible binding to FXR. The binding affinities of alisol C 23-acetate and alisol B 23-acetate to FXR were similar. As shown in Supplementary Figure S1, alisol B 23-acetate was sterically compatible with the binding site of FXR and formed hydrogen bonds via the oxygen atom in the ring ether at C-24 with the side chain N atom of His294 and Arg331 in FXR. Other hydrogen bonds were observed between the C-11 hydroxyl groups and the backbone O atom of Leu287 and the O atom in the carbonyl groups with the side chain N atom of His447 and the side chain O atom of Tyr361. Van der Waals (VDW) interactions were also observed between alisol B 23-acetate and protein FXR, which likely contributed to the binding energy.

As shown in Supplementary Figure S2, the binding mode of alisol C 23-acetate with FXR protein was similar to that of alisol B 23-acetate, except the absence of the hydrogen bond with His294. Alisol B had relatively poor affinity to FXR with a docking score of −10.38 kcal/mol and formed hydrogen bonds with Arg331, His447, and Tyr361 (Supplementary Figure S3). Overall, alisol B 23-acetate, alisol C 23-acetate, and alisol B can bind to FXR via the hydrogen bond and VDW interactions. The results of docking suggested the three core active compounds might present the lipid-lowering effect by affecting the activity of FXR.

## 4. Discussion

TCM formulas are promising in treating complex pathologies for their characteristics of multicomponent and multipathway. Their extremely complex chemical composition, however, poses challenges to quality control. Thus, TCM formulas can hardly be broadly accepted by the international medical community, especially the western academia [37]. Spectrum-effect study is considered as an effective technique to find active ingredients in TCM and contributes a lot for the quality control [38]. The premise of performing spectrum-effect analysis is that there must be a certain amount of samples to supply the diversity chemical spectrums and pharmacodynamic information. For the single Chinese herbal medicine, different varieties, regions, and different harvest times all contribute to provide sufficient samples with different material basis for the analysis [8]. When it comes to TCM prescription, screening simple and appropriate methods to guarantee the required samples is an imperative and challenging step needed to be overcome. In the previously reported literature, the changing therapeutic effects brought about by altering the ratio of AR and AMR from 5 : 2 to 2 : 5 or 1 : 1 were memorably observed [39]. This result yielded a creative hint to us that it might be feasible to conduct the spectrum-effect study from the perspective of changing the compatibility of AR and AMR in ARD. Therefore, we made a new attempt by setting the ratio of AR and AMR as 3 : 1, 5 : 2, 2 : 1, 3 : 2, 1 : 1, 2 : 3, 1 : 2, 2 : 5, and 1 : 3 to gain dissimilar ARD samples for the analysis, and our result proved this idea could be successfully applied into the spectrum-effect analysis and was easy to be operated to explore pharmacological ingredients in ARD. Specifically, qualitative trends (positive or negative) were obtained through PLSR [31], and quantitative explanations were from GRA and VIP values [16, 40]. Peaks of X20 (alisol B 23-acetate), X9 (alisol C 23-acetate), and X18 (alisol B) showed positive correlation coefficients in Figure 3, and they got high VIP values as well as grey relational coefficients compared with other peaks at the same time. Accordingly, the three ingredients were more likely to have better lipid-lowering efficiency than others in ARD. Therefore, our work offered valuable insights into the research of efficacy components in TCM formula such as ARD. Furthermore, to ensure the accuracy of the spectrum-effect analysis, the chromatographic and mass spectrometric conditions were optimized, and the water content in AR and AMR was determined using near-infrared spectroscopy [41].

ARD has been shown to be of clinical value in treating hyperlipidemia. The lipid-lowering effect of ARD is generally considered to correspond with a flurry of triterpenoids and lactones present in ARD, such as alisol A, alisol B, alisol A 23-acetate, alisol A 24-acetate, and alisol B 23-acetate [6, 42–44]. Related investigations, however, focused more on the lipid-lowering effect of monomeric compounds. The monomeric compounds were administered *in vivo* and *in vitro*, which was not equivalent to administration of TCM formulas for different contents of various compounds, let alone their combinations. Thus, the core lipid-lowering components present in the prescription ARD are actually unclear.

In previous studies, a large number of compounds were identified in AR and AMR [35, 45], which could contribute greatly to their quality control and pharmacological research. However, identification of compounds in ARD was scanty. It was reported that alisol A, alisol B, alisol B 23-acetate, atractylode I, atractylode II, and atractylode III were determined in ARD [46]. Moreover, we determined twenty-four compounds in the ethanol and aqueous extracts of ARD [21]. Basing on these results, we further identified twenty common peaks in ARD, of which 15 compounds were of definitive structure. Alisol B 23-acetate, alisol C 23-acetate, and alisol B were considered to be the core lipid-lowering components of ARD through the mathematical models of the spectrum-effect study. Molecular docking further showed that these compounds can bind to the FXR protein through hydrogen bonding and VDW interactions, and the key binding sites were the carbonyl group at C-3, hydroxyl groups at C-11, and the ring ether structure at C-24. A previous study showed that alisol B 23-acetate reduced hepatic lipogenesis and increased lipolysis via FXR activation, resulting in anti-inflammatory, anti-fibrotic, and hepatoprotective effects [17, 47]. Thus, it is rational to surmise that alisol B and alisol C 23-acetate may also exert their lipid-lowering effects as FXR agonists. Since alisol C 23-acetate exhibited almost the same binding affinity to FXR as alisol B 23-acetate, its biological activity is worth further exploration.

FXR regulates cholesterol and triglyceride metabolism and is therefore an attractive therapeutic target for hyperlipidemia. Based on our findings, it is possible that ARD inhibits lipid accumulation by blocking FXR-regulated factors involved in lipid metabolism, including cholesterol 7*α*-hydroxylase 1 (CYP7A1), lipoprotein lipase (LPL), peroxisome proliferator-activated receptor *α* (PPAR*α*), carboxylesterase 1 (CES1), and sterol regulatory element-binding protein-1c (SREBP-1c) [48–50]. Furthermore, given the role played by FXR in nonalcoholic fatty liver disease (NAFLD) [51], ARD is also a promising pharmacological agent against NAFLD.

## 5. Conclusion

In the present study, the relationship between the spectrum and efficacy of 9 samples of ARD with different ratios of AR and AMR was elucidated. With the help of chemometric methods, alisol B 23-acetate, alisol C 23-acetate, and alisol B were found might be the core bioactive components of ARD. The three compounds can bind to the FXR protein via hydrogen bonding and VDW interactions, indicating that they may exert lipid-lowering effects as FXR agonists. This study provided new impetus for the exploration of the active constituents and further molecular mechanism studies of TCM such as ARD.

## Figures and Tables

**Table 1:** The areas of the common peaks in 9 ARD samples.

Common peaks	Peak areas in 9 ARD samples								
	S1	S2	S3	S4	S5	S6	S7	S8	S9
X1	996.49	772.03	802.99	678.26	750.86	517.12	573.22	443.79	666.65
X2	576.24	461.66	543.27	438.50	449.47	360.47	362.27	310.59	375.40
X3	633.39	440.18	710.22	992.83	1975.50	1478.83	2471.61	2489.34	3928.51
X4	2029.60	1762.89	1985.89	1469.20	1977.12	1527.29	1649.38	1833.18	1391.95
X5	294.70	350.64	354.39	265.54	473.93	550.52	863.18	1141.33	692.75
X6	6171.31	3689.06	4215.43	2392.49	3419.99	1488.73	2242.15	2071.95	2113.89
X7	516.04	576.06	663.64	781.79	1313.59	1650.60	1667.32	2645.09	1682.63
X8	789.88	786.46	349.65	539.43	1149.06	1352.83	1603.67	2013.26	1973.99
X9	11087.01	9100.50	8631.49	6072.15	9772.90	13245.24	10478.00	15195.76	20724.83
X10	4105.11	3858.75	2963.54	2122.15	2677.13	1673.27	1218.11	1458.11	1100.20
X11	5590.23	5009.76	5143.05	4590.42	7865.31	9479.26	8047.92	11133.95	14200.69
X12	1000.24	1183.13	2134.83	567.92	1318.51	1643.56	1248.90	583.34	922.58
X13	1526.39	873.43	875.00	983.19	990.28	435.68	674.90	696.39	390.59
X14	1845.03	1559.67	1449.25	975.59	1502.72	1352.89	1323.91	1266.34	1039.21
X15	2872.41	2502.48	1888.94	1054.12	2614.97	1694.21	2792.14	1480.87	1223.28
X16	459.78	749.41	969.85	663.81	1401.53	1322.90	953.93	1004.26	1115.96
X17	696.37	525.79	593.17	478.91	748.75	478.68	403.52	397.15	324.80
X18	14094.98	15223.77	16909.15	13409.98	15985.27	12960.30	11928.73	9230.19	8520.34
X19	418.56	288.49	395.31	257.13	982.28	1186.61	1907.50	1545.04	2273.31
X20	17477.60	18645.05	17448.52	13568.44	17910.34	12804.03	12882.97	12590.62	9812.31

**Table 2:** Identification of the common peaks.

Peak	t_R__(min)_	MS^1^ [M+H]^+^	Error (ppm)	Fragment ions collected in positive mode	Molecular formula	Identification
1	34.88	471.2136	1.1	453.2013, 295.1641, 277.1545, 248.1306, 177.0552, 145.0550	C_25_H_31_N_2_O_7_	Unknown
2	35.55	469.1962	−2.8	293.1486, 276.1258, 219.1372, 177.0576, 145.0312	C_25_H_29_N_2_O_7_	Unknown
3	37.76	389.2546	1.8	371.2191, 330.2155, 284.1887, 244.1507	C_20_H_36_O_7_	Tetradecylcitric acid
4	39.72	203.1785	−4.6	161.1245, 147.1129, 133.0995, 119.0852	C_15_H_22_	Atractylenolide VI
5	40.73	487.3411	−2.5	469.3322, 451.3216, 415.2839, 397.2657	C_30_H_46_O_5_	Alisol C^a^
6	41.24	487.3425	0.4	469.3325, 451.3220, 397.2732	C_30_H_46_O_5_	16-oxo-11-anhydro-alisol A
7	41.65	274.1809	0.7	177.0571, 145.0363	C_17_H_24_NO_2_	Unknown
8	42.26	230.1539	−2.6	159.1148	C_15_H_19_NO	Atractylenolactam
9	43.25	529.3553	4.5	511.3440, 469.3327, 451.3228, 415.2849, 397.2732	C_32_H_48_O_6_	Alisol C 23-acetate^a^
10	43.69	469.3319	0.2	451.3215, 397.2744	C_30_H_44_O_4_	Alisol L
11	44.06	233.1543	0.4	215.1426, 187.1472, 177.1213, 159.1075, 145.0978	C_15_H_20_O_2_	Atractylenolide I^a^
12	44.28	473.3618	−3.8	455.3517, 437.3402, 383,2941, 365.2842, 339.2682	C_30_H_48_O_4_	Alisol G
13	44.88	494.3636	0.4	453.3376, 381.2792, 339.2673	C_32_H_48_NO_3_	Unknown
14	46.24	545.3496	3.3	527.3380, 485.3280, 467.3181	C_32_H_48_O_7_	Alisol M 23-acetate
15	46.56	513.3589	1.8	495.3184, 453.3332, 435.3245, 381.2845	C_32_H_48_O_5_	Alisol O
16	46.92	455.351	−3.3	437.3420, 419.3304, 383.2925, 365.2813, 339.2660	C_30_H_46_O_3_	Alisol I
17	47.15	511.3437	2.7	493.3347, 451.3136, 433.2851	C_32_H_46_O_5_	Alisol L 23-acetate
18	47.44	473.3639	1.7	455.3535, 437.3431, 383.2944, 365.2848, 339.2687	C_30_H_48_O_4_	Alisol B^a^
19	48.68	499.2791	2.0	455.3461, 423.3522, 365.2821, 295.1573, 2111.0988	C_32_H_39_N_2_O_3_	Unknown
20	49.49	515.376	4.7	497.3654,4 79.3536, 437.3434, 419.3329, 383.2957, 365.2858, 339.2960	C_32_H_50_O_5_	Alisol B 23-acetate^a^

^a^Compared with the reference standards.

**Table 3:** Cell viability and effect on lipid accumulation in HepG_2_ cells of 9 ARD samples(Mean ± SD, *n* = 6).

Samples	Ratio of AR and AMR	Cell viability (%)	Fluorescence intensity
1	3 : 1	95.41 ± 10.94	39514.50 ± 3448.72
2	5 : 2	99.05 ± 19.17	39541.50 ± 1661.85
3	2 : 1	91.20 ± 4.63	34849.00 ± 1807.24
4	3 : 2	91.04 ± 1.94	42702.83 ± 795.48
5	1 : 1	93.64 ± 14.77	39551.67 ± 5542.87
6	2 : 3	96.04 ± 6.99	47602.67 ± 2407.80
7	1 : 2	91.79 ± 6.93	45362.83 ± 3745.42
8	2 : 5	95.99 ± 7.60	45707.33 ± 3578.40
9	1 : 3	91.66 ± 14.99	41840.50 ± 3493.73
Blank group	—	100.00 ± 2.57	8966.00 ± 197.55
Model group	—	99.81 ± 2.33	44818.00 ± 1796.97

## Data Availability

The original contributions presented in the study are included in the article/supplementary material; further inquiries can be directed to the corresponding author.

## Supplementary Materials

Supplementary Materials The details of docking, including docking scores and binding models, are provided under the supplementary section.
