# Supplementary material for: A Strategy for Screening the Lipid-Lowering Components in Alismatis Rhizoma Decoction Based on Spectrum-Effect Analysis
Source: J Anal Methods Chem. 2022 Jan 4;2022:2363242. doi: 10.1155/2022/2363242 (PMC8752264; doi:10.1155/2022/2363242)
Supplement: Supplementary Materials — The details of docking, including docking scores and binding models, are provided under the supplementary section. [file 2363242.f1.doc]

**Table S1.** The docking scores of ligand compounds with protein FXR.

| **Ligand** | **Receptor** | **Docking score (kcal/mol)** |
| --- | --- | --- |
| Alisol B 23-acetate | FXR | -11.12 |
| Alisol C 23-acetate |  | -11.07 |
| Alisol B |  | -10.38 |

**Figure S1. A:** The 2D binding mode of alisol B 23-acetate and FXR. **B:** The binding model of alisol B 23-acetate on molecular surface of FXR. **C:** The 3D binding mode of alisol B 23-acetate and FXR.


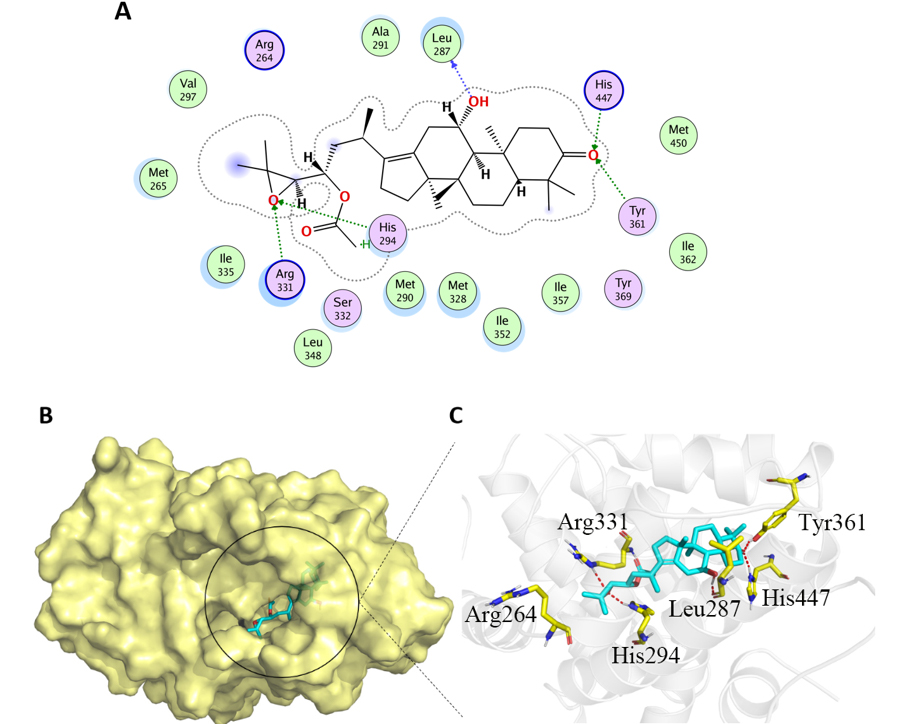


**Figure S2. A:** The 2D binding mode of alisol C 23-acetate and FXR. **B:** The binding model of alisol C 23-acetate on molecular surface of FXR. **C:** The 3D binding mode of alisol C 23-acetate and FXR.


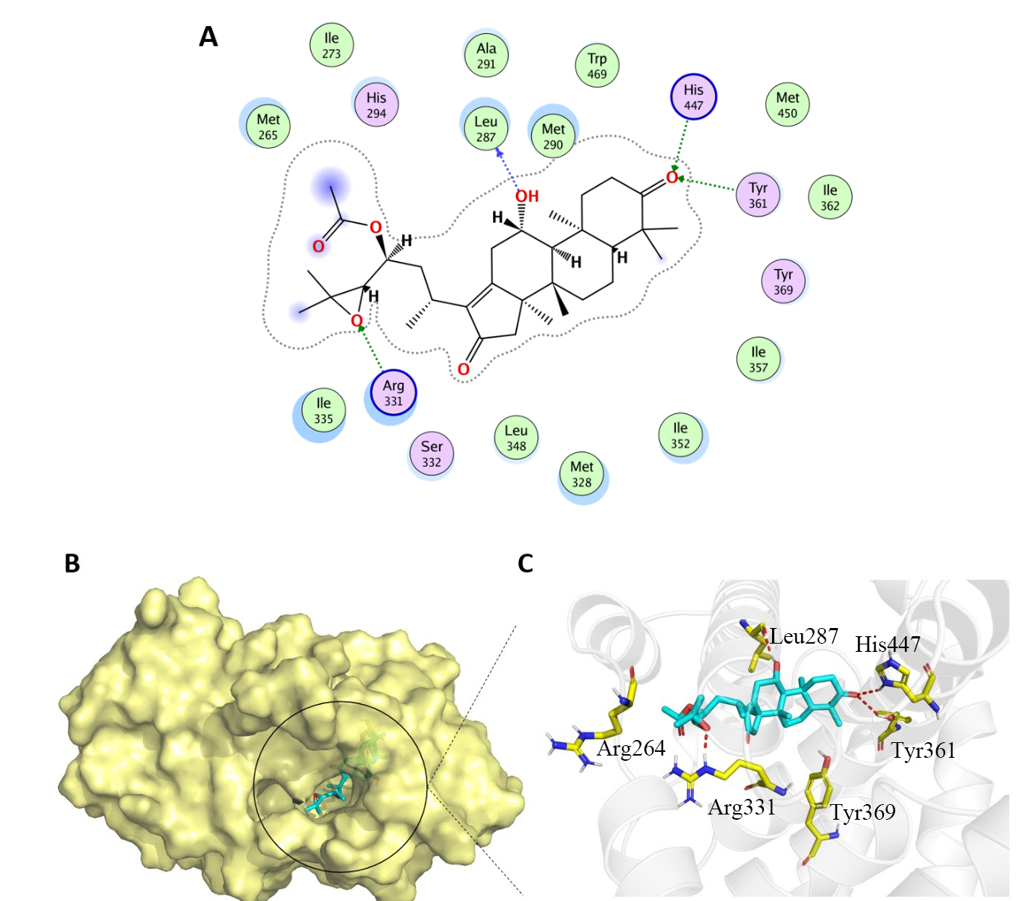


**Figure S3. A:** The 2D binding mode of alisol B and FXR. **B:** The binding model of alisol B on molecular surface of FXR. **C:** The 3D binding mode of alisol B and FXR.

**
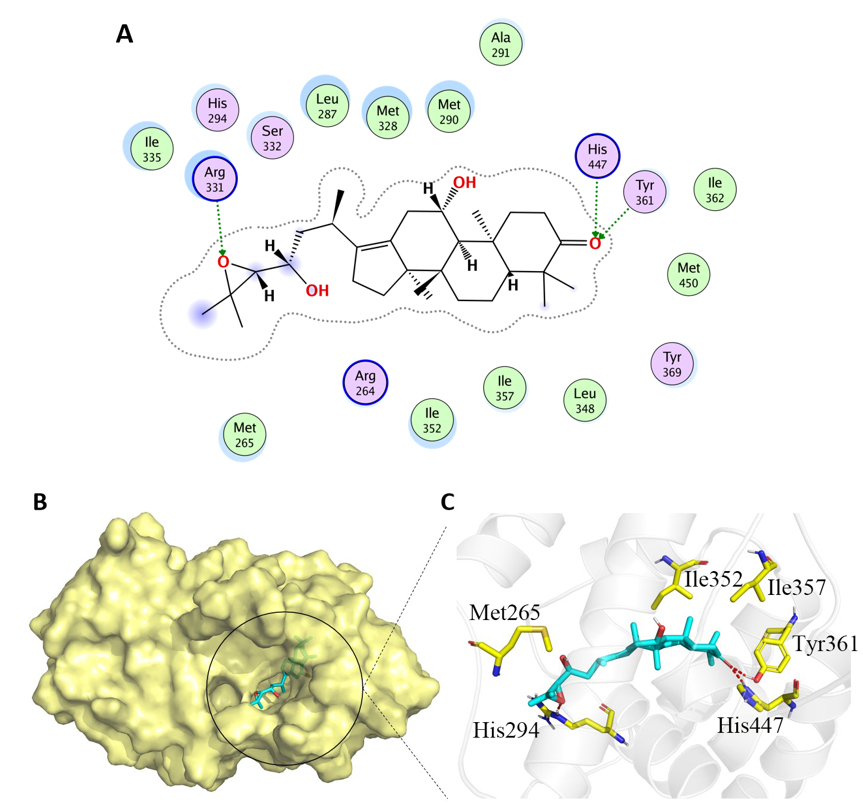
**
